# Editorial: Nutraceuticals for the recovery of COVID-19 patients

**DOI:** 10.3389/fnut.2022.1054632

**Published:** 2022-11-14

**Authors:** Tilakavati Karupaiah, Kuo-Cheng Lu

**Affiliations:** ^1^Faculty of Health and Medical Sciences, School of BioSciences, Taylor's University, Subang Jaya, Selangor, Malaysia; ^2^Division of Nephrology, Department of Medicine, Taipei Tzu Chi Hospital, Buddhist Tzu Chi Medical Foundation, New Taipei City, Taiwan; ^3^Division of Nephrology, Department of Medicine, School of Medicine, Fu Jen Catholic University Hospital, Fu Jen Catholic University, New Taipei City, Taiwan

**Keywords:** COVID-19, dietary patterns, nutraceuticals, vegetarian diet, vitamin D

The novel coronavirus disease (COVID-19) unleashed sudden and unprecedented mortality on global populations and fosters a lingering health burden. In our call for papers on the theme ***Nutraceuticals for the recovery of COVID-19 patients***, we purposively invited topics on the immunomodulatory effects of nutrients and bioactive compounds falling into the narrow definition of nutraceuticals and functional foods (1–3) as well as dietary supplements and designed diets (4), given the knowledge gaps in adjunctive therapy management for the post-infection stages of COVID-19. Falling within this research theme are 10 papers covering dietary protein (Shariatpanahi et al.), melatonin (Su et al.), curcumol (Yang et al.), herbal tea (Hsieh et al.), dietary supplements (Hashemi et al.), and dietary patterns (Ebrahimzadeh et al.; Hou et al.; Vajargah et al.) as well as vitamin D (Bogliolo et al.; Chiang et al.) (Figure 1).

**Figure 1:**
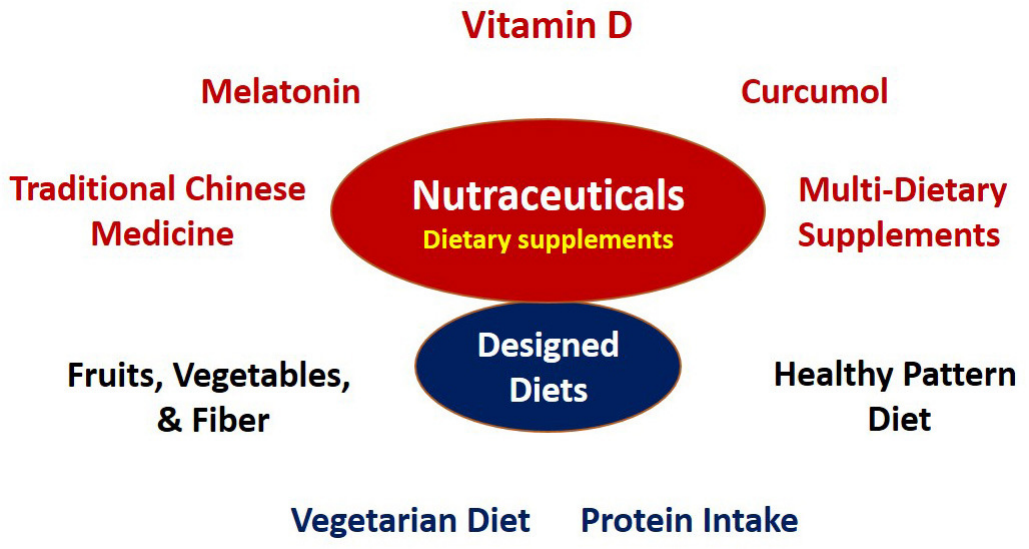
Nutraceutical supplements and various dietary patterns for the recovery of COVID-19 patients.

Malnutrition is prevalent in COVID-19-infected patients, particularly in those with a greater severity of the disease and who are critically ill (1). A major complication associated with the initiation of feeding in malnourished patients is refeeding syndrome (2). In a prospective cohort study, Shariatpanahi et al. assessed patients for their risk of developing refeeding syndrome and those who did develop it. They found the incidence of refeeding syndrome was relatively high in the majority of critically ill COVID-19 patients, but increased protein intake was associated with reduced occurrence of refeeding syndrome.

The protective role of vitamin D in COVID-19 sufferers (3) is commonly researched for its immunomodulatory and anti-inflammatory action at the level of endothelial function (4–6), and is highly recommended as an adjuvant therapy for COVID-19 (7). In this special issue, a prospective observational multicenter study by Bogliolo et al. showed that very low 25(OH) vitamin D levels were highly prevalent in patients with severe COVID-19, but low 25(OH) vitamin D levels were not associated with high mortality outcomes in moderate to severe cases of COVID-19. This finding is contrary to a meta-analysis of seven systematic reviews (7) that showed that vitamin D supplementation reduces the risk of mortality, need for intensive care, and mechanical ventilation requirements in COVID-19 patients. Another direction in vitamin D adjuvant therapy is in kidney disease, given the concern that COVID-19 patients who are asymptomatic or have mild symptoms show dynamic changes in renal function (8), whilst patients with chronic kidney disease (CKD) frequently have vitamin D deficiency and increased susceptibility to infection. In their review article, Chiang et al. highlight the double burden of increased risk for vitamin D deficiency in CKD patients due to the coexistence of immune activation and immune deficiency, and proposed mechanisms by which vitamin D administration could modulate the immune system and alleviate the pathological consequences of COVID-19. A further benefit of vitamin D supplementation would be to reduce the severity of acute kidney injury in COVID-19 patients *via* reducing soluble urokinase-type plasminogen activator receptor levels.

Factors such as age, sex, and comorbidities are key determinants of illness severity and progression of COVID-19. The review article by Su et al. centers attention on the decline in melatonin levels exacerbated by aging, with a strong implication of compromised mitochondrial redox activities which could explain the higher death rate of COVID-19 in older age groups. Declining melatonin levels are closely related to mitochondrial dysfunction, and its reversal with melatonin supplementation could limit virus-related diseases. Hence, melatonin in elderly people may be warranted in the treatment of COVID-19.

The special edition introduces curcumol as a common traditional Chinese medicine (TCM), isolated from Rhizoma Curcumae with well-documented anti-viral activity (9). By using network pharmacology and systematic bioinformatics analysis, Yang et al. identified seven core targets of curcumol therapy for lung adenocarcinoma (LUAD) patients infected with COVID-19. These targets influence cell-signaling associated with the Warburg effect, which supports SARS-CoV-2 replication and inflammatory response. Comparative transcriptomic analysis specified the effects of curcumol through control of cell cycle, DNA damage response, and cell apoptosis. The combination of TCM and standard management in treating patients with COVID-19 in Taiwan was examined using Jing Si Herbal Tea (JSHT). A prospective cohort study by Hsieh et al. that recruited patients with mild to moderate COVID-19 suggests JSHT combined with standard management may prevent critical status and mortality. Effective improvements in measured outcomes such as reverse transcription–polymerase chain reaction cycle threshold value, C-reactive protein level, and Brixia score occurred in male and older patients (≥60 years), suggesting that three main pathophysiological pathways, anti-infective, anti-inflammation, and anti-thrombosis, were potentially targeted (10).

The ability of purposive dietary patterns to protect against respiratory viral infections and reduce associated inflammation and oxidative stress is also examined in this special edition. A retrospective evaluation of COVID-19 patients by Hou et al. found COVID-19 symptom severity was significantly and inversely associated with adherence to a self-reported vegetarian diet compared to those consuming a non-vegetarian diet, with the latter group having a higher risk in contracting critically severe COVID-19. A cross-sectional study of COVID-19 hospitalized patients by Vajargah et al. showed higher consumption of fruits, vegetables, and fiber was inversely linked with COVID-19 severity, clinical symptoms, hospitalization, and convalescence duration, and concentrations of inflammatory markers. Fruits and vegetables are rich in fiber and a good source of anti-inflammatory and immune-boosting vitamins, minerals, and antioxidants (11). In contrast, the pre-COVID-19 status of habitual food intake could be an environmental factor affecting inflammation status in the body (12) and potentiate outcome response to COVID-19 infection (13). Ebrahimzadeh et al. retrospectively evaluated 250 recovered COVID-19 cases to explore diet pattern effects using a self-reported web-based food questionnaire. They found cases reporting a higher adherence to a healthy diet pattern were associated with lower inflammatory markers levels and lower risk of COVID-19 severity, hospitalization, and convalescence duration.

Was consumption of immune-boosting supplementation critical to offering protection during the COVID-19 pandemic? Hashemi et al. in a cross-sectional study involving 300 adult men and women with COVID-19, probed recent and long-term supplement intakes using a questionnaire. Short-term use (~2 months) saw improvements in blood urea nitrogen and higher serum 25(OH)D levels whilst long-term use achieved significantly lower invasive oxygen support, lactate dehydrogenase (LDH), fewer days of fever, and higher serum 25(OH)D levels.

The papers included under the theme of *Nutraceuticals for the recovery of COVID-19 patients* are highly relevant to the emergence of long COVID symptoms as a health burden and the need for encouraging more research in this area.

## Author contributions

KC-L wrote the introduction and the conclusion. TK wrote the central part with comments on the cited papers and references. Both authors contributed to the article and approved the submitted version.

## Conflict of interest

The authors declare that the research was conducted in the absence of any commercial or financial relationships that could be construed as a potential conflict of interest.

## Publisher's note

All claims expressed in this article are solely those of the authors and do not necessarily represent those of their affiliated organizations, or those of the publisher, the editors and the reviewers. Any product that may be evaluated in this article, or claim that may be made by its manufacturer, is not guaranteed or endorsed by the publisher.
